# ‘Follow the Water’: Microbial Water Acquisition in Desert Soils

**DOI:** 10.3390/microorganisms11071670

**Published:** 2023-06-27

**Authors:** Don A Cowan, S. Craig Cary, Jocelyne DiRuggiero, Frank Eckardt, Belinda Ferrari, David W. Hopkins, Pedro H. Lebre, Gillian Maggs-Kölling, Stephen B. Pointing, Jean-Baptiste Ramond, Dana Tribbia, Kimberley Warren-Rhodes

**Affiliations:** 1 Centre for Microbial Ecology and Genomics, Department of Biochemistry, Genetics and Microbiology, University of Pretoria, Pretoria 0002, South Africa; pedro.lebre@up.ac.za (P.H.L.); jbramond@bio.puc.cl (J.-B.R.); 2 School of Biological Sciences, University of Waikato, Hamilton 3216, New Zealand; craig.cary@waikato.ac.nz; 3 Departments of Earth and Biology and Planetary Sciences, Johns Hopkins University, Baltimore, MD 21218, USA; jdiruggiero@jhu.edu; 4 Department of Environmental and Geographical Science, University of Cape Town, Cape Town 7701, South Africa; frank.eckardt@uct.ac.za; 5 School of Biotechnology and Biological Sciences, University of New South Wales, Sydney, NSW 2052, Australia; b.ferrari@unsw.edu.au (B.F.); d.tribbia@student.unsw.edu.au (D.T.); 6 Scotland’s Rural College, West Mains Road, Edinburgh EH9 3JG, UK; david.hopkins@sruc.ac.uk; 7 Gobabeb-Namib Research Institute, Walvis Bay 13013, Namibia; gillian@gobabeb.org; 8 Department of Biological Sciences, National University of Singapore, Singapore 117558, Singapore; yncpsb@nus.edu.sg; 9 Departamento Genética Molecular y Microbiología, Pontificia Universidad Católica de Chile, Santiago 7820436, Chile; 10 NASA Ames Research Center, Moffett Field, CA 94035, USA; kim_lamma@yahoo.com

**Keywords:** anhydrobiosis, desert soils, hyper-arid, microbiomes, desiccation, xerophily, moisture stress, water activity, water availability

## Abstract

Water availability is the dominant driver of microbial community structure and function in desert soils. However, these habitats typically only receive very infrequent large-scale water inputs (e.g., from precipitation and/or run-off). In light of recent studies, the paradigm that desert soil microorganisms are largely dormant under xeric conditions is questionable. Gene expression profiling of microbial communities in desert soils suggests that many microbial taxa retain some metabolic functionality, even under severely xeric conditions. It, therefore, follows that other, less obvious sources of water may sustain the microbial cellular and community functionality in desert soil niches. Such sources include a range of precipitation and condensation processes, including rainfall, snow, dew, fog, and nocturnal distillation, all of which may vary quantitatively depending on the location and geomorphological characteristics of the desert ecosystem. Other more obscure sources of bioavailable water may include groundwater-derived water vapour, hydrated minerals, and metabolic hydro-genesis. Here, we explore the possible sources of bioavailable water in the context of microbial survival and function in xeric desert soils. With global climate change projected to have profound effects on both hot and cold deserts, we also explore the potential impacts of climate-induced changes in water availability on soil microbiomes in these extreme environments.

## 1. Introduction

“Where there is water, there is life”. This concept is believed to be almost universally true and has been a guiding principle in the search of past, and even extant, life on other planets [1,2,3]. Understanding how, and from where, microorganisms in xeric habitats can acquire water to support survival and metabolic activity is critical in understanding the functional ecology of Earth’s drylands and how their soil microbiomes may respond to the effects of global climate change.

Water availability is widely accepted as the dominant driving force for the structure and function of microbial communities in both hot and cold desert soils [4,5,6,7,8]. Much microbial life on Earth cannot undergo cell division below a water activity of approx. 0.900, which is equivalent to a relative humidity of 90.0% [8,9]. Similarly, most microorganisms also cannot exhibit higher-order metabolic functions (such as respiration or photosynthesis) under conditions that are dryer than the lower water-activity value for cell division [10]. The exceptions are a small number of xerophilic or halophilic taxa that possess functional adaptations supporting cellular growth functions at lower water activity levels [8,10]. The apparent constraints of low water availability notwithstanding, even the most hyper-arid hot deserts, such as in the Atacama, the Namib, and the Sahara, harbour substantial populations of viable and functional microorganisms in soils and lithic niches [11,12,13,14].

However, many other parts of the biosphere, including Earth’s subsurface and atmosphere, may exist, permanently or transiently, in a state of limited water activity. Much of the Earth’s atmosphere, which represents a large-scale repository of microbial life [15,16], has a relative humidity of less than 80% [17].

The mandatory requirement for water is more exacting, however, and should probably be rephrased as ‘where there is liquid water, there is life’. We acknowledge notable exceptions, such as the super-heated emission fluids of deep-sea hydrothermal vents [18] where temperatures are much too high (>>120 °C) to sustain life. Those few terrestrial habitats where water is present but never (or very rarely) in a liquid form, such as the extreme polar deserts [19,20,21], harbour few (or possibly no) viable microorganisms. The apparent anomaly may be permafrost where, despite permanently frozen conditions, substantial populations of viable microorganisms exist [22], and there is solid evidence for in situ metabolic activity [23,24]. The possibility of micro-scale solute-rich liquid inclusions in permafrost horizons, containing metabolically active microorganisms, has been considered [25], but has not been definitively demonstrated.

There is also recent evidence that dry surfaces may acquire thin films or micrometre-sized droplets of water [26] that can support microbial survival during periods of desiccation. While this process has only been demonstrated on leaf surfaces and is driven by the deliquescence of aerosol-derived hygroscopic particles [27], it is not unreasonable to assume that similar processes may occur on rock surfaces, particularly where marine aerosols could provide a steady input of hygroscopic marine salts. This process might be of particular significance as a source of water input to exposed rock surfaces in coastal deserts, such as the Namib [28] and Atacama [29,30].

Given the current trends in global warming and the predictions of expanding deserts and drylands [31], we believe that developing understanding of complex interactions between soils, microbiomes, and the hydrological cycle is particularly important. Understanding both the qualitative and quantitative aspects of microbial water acquisition in desert soils may result in a re-evaluation of the functional roles, and ultimately, the ecosystem services, of desert soil microbiomes.

Here, we examine the potential sources of water for desert-soil microorganisms, the mechanisms by which microbial cells may access water, and the implications of global climate change for desert soil microbial communities.

## 2. Desert Soils and Their Water-Holding Capacity

Soils in deserts are typically classified within 5 of the 32 major soil groups in the FAO’s World Reference Base for soils [32]; however, there is no single group that exclusively covers desert soils. The five most common soils in deserts are Arenosols, Leptosols, Cambisols, Calcisols, and Solonchaks. Arenosols are sandy soils that are usually easily eroded and have low water- and nutrient-holding capacities, typical of coastal and inland dunes and sand seas. Leptosols are thin soils over rock or gravel. Cambisols are relatively young soils in terms of pedogenic development (although more developed that Arenosols and Leptosols). Calcisols are soils with accumulations of calcium carbonate usually precipitated from the soil water due to evaporation at the surface. Solonchaks are soils in arid regions with concentrations of salts due to evaporation (in contrast to highly salt-containing soils in coastal regions). Combinations of limited water and nutrient contents, shallowness, impenetrable hard layers, and high osmotic potential typically lead to limited water accessibility, restricting soil biological activity.

The distribution of water in a porous soil matrix is thermodynamically driven by a maximisation of the surface interactions with the internal surfaces of pores and voids. The surface interactions, including capillarity, lead to the narrowest pores becoming water-filled first when soil is gradually wetted, followed progressively larger pores. The water potential (effectively the force with which water is held in the soil pore space) is the combination of the matric effects (e.g., pore size distribution, pore connectivity, and neck size constrictions), pressure, humidity, gravity, and solute pressures, where these combined forces must be overcome for effective water extraction by plant roots or microorganisms. Under conditions of extreme dryness, liquid water is held only in the smallest soil pores and may therefore be inaccessible to soil microorganisms [33]. However, for many desert soils, where the sand-sized fraction predominates, pore distribution is dominated by larger pores because of the lower packing density of sand particles, and therefore, smaller forces acting to retain water; thus, physical accessibility is less of a restriction than the actual scarcity of water.

## 3. Sources of Bioavailable Water in Desert Ecosystems

### 3.1. Rainfall

**Figure 1:**
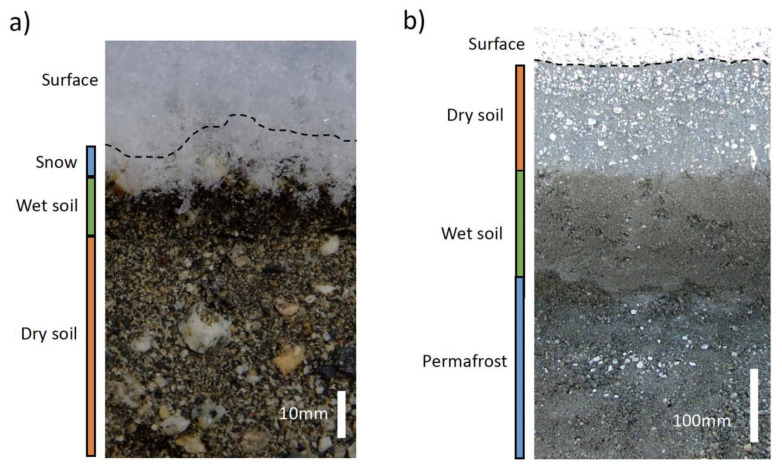
(**a**) Moist surface horizon (approx. 10 mm) from snowmelt (Miers Valley, East Antarctica, January 2012); (**b**) soil horizon profile, showing dry surface mineral soils, moistened active horizon, and frozen (permafrost) zone (Miers Valley, East Antarctica, January 2008).

Rainfall is the most obvious source of liquid water for Earth’s terrestrial ecosystems. However, some hot deserts experience decadal periods between precipitation events [34], and some extremely cold deserts, such as the Antarctic McMurdo Dry Valleys, very rarely receive rainfall [35]. Precipitation events in the latter are in the form of occasional snowfall, where much of the settled snow does not melt but is lost back to the atmosphere via sublimation [36]. Surface snowmelt may only wet the upper 0.5–1.0 cm of soil (Figure 1 in [37]; Cowan, unpublished observations: see Figure 1a). Snow deposition on dry mineral surfaces is thought to provide water for shallow subsurface (2 to 5 mm depth) cryptoendolithic microbial communities [38].

The extent to which, and duration for which, soils retain water after precipitation is highly relevant to the capacity for soil microbiome functionality. Where present on desert soil surfaces, biological soil crusts (BSCs) are important in retaining precipitation that would otherwise be lost by evaporation or rapid infiltration to subsurface soil [39]. Both soil structure and composition are important in water retention [40] where, for example, clays such as sepiolite, palygorskite, and smectite, often associated with arid soil environments and evaporitic rock substrates, exhibit a very high water-holding capacity (250% of wt [16,41,42]). Organic substances in soils, such as plant biomass, humic acids, and particularly microbial extracellular polymeric substances (EPSs), are very hygroscopic. Water is less readily lost to evaporation or filtration in saline soils [43] and, while water in saline soils is generally considered to be less available for plant uptake, the hygroscopic matrices of microbial biofilms may compete effectively for salt- and clay-bound water [44].

Although long-term precipitation patterns in hyper-arid deserts, coupled with very high evaporation rates, may suggest that persistent microbial communities are not sustainable [45], there is clear evidence that specialized niches in below-ground soils and endoliths offer ephemeral microbial habitats after significant stochastic rainfall episodes [14,46,47,48].

### 3.2. Glacial Ice

Icecaps and other glacial masses might be considered as desert-like ecosystems, in that the consistently low temperatures typically characterizing all but the surface horizons of such environments can ensure that water is rarely, if ever, present in bioavailable liquid form. However, liquid water can exist on both the upper and lower surfaces of many glacial masses. For example, biologically active surface microbial communities are associated with the localized melting of many glacial masses, snow algae and cryoconite communities being two well-known examples. Both communities acquire liquid water from the frozen snow/ice substrate through the same mechanism: a reduction in albedo (reflected incident light) corresponding to an increase in the absorption of solar radiation (solar gain) [49]. In snow and ice algae (mostly *Chlamydomonadales* and *Zygnematales*, respectively), astaxanthin- and purpurogallan-rich pigmented cells absorb solar radiation with an associated heat-gain that melts frozen water (snow/ice) in the immediate vicinity of the cells [49]. Similarly, cryoconite holes are created by the deposition of dust particles and rock fragments/pebbles on snow and ice surfaces. The dark mineral particles are warmed by the absorption of solar radiation, and generate melt-wells in the ice surface, which rapidly become rich oases of metabolically active microbial life [50]. On the undersides of glacial masses, heat, generated by friction between the ice mass and the underlying rock surface, generates meltwater; sub-glacial liquid systems support substantial microbial populations [51].

### 3.3. Dew and Fog

In desert soil ecosystems, water may be available in the form of dew or fog inputs. Dew formation results from a balance between atmospheric relative humidity and temperature, where condensation occurs on surfaces when the balance exceeds the ‘dew point’ [52]. Dewfall is a relatively common occurrence in many hot semi-arid and arid (but not hyper-arid) deserts (up to 200 days per annum in some Negev Desert locations [53]) and is likely to make a significant contribution to the water input budgets of the surface (0–1 cm depth) soils [54] and their microbiomes. Dew water input in the Badain Jaran Desert (northwest China) over a 5-month period amounted to a total of 3.4 mm, averaging 0.06 mm d^−1^ [55]. Dew is thought to be an important supplementary water source for desert vegetation. Soil surface lichens and surface microbiological communities (BSCs) have been shown to benefit from dew water inputs [56,57,58]. In the Atacama Desert, characteristic microkarstic features found on the surface layers of calcite rocks indicated that dew deposition might be an important source of liquid water for the endolithic communities inhabiting the calcite rock [30].

Fog water inputs are restricted to a limited number of coastal desert ecosystems, most notably the Namib (Namibia, south-western Africa) and Atacama (north-west Chile) deserts [34,59]. In the Namib Desert, fog generated off-shore by moist air over the Benguela Current is driven inland at night by onshore wind-flows, and can penetrate inland up to around 60 km [34]. At the coast, fog events are frequent (est. 40% events per annum [60]) and sustain extensive and well-characterized lichen fields [61]. This conclusion was recently supported by an extensive remote sensing drone survey of coastal Namib Desert lichen fields using advanced photogrammetry, which showed that *Xanthoparmelia* and *Stellanrangia* spp. preferentially colonized ocean-facing rock surfaces, i.e., the direction from which fog originates [62].

Further inland, water capture from less frequent fog events sustains both specialist plants (e.g., Speargrass (*Stipagrostis sabulicola*) [63] and insect species (e.g., dune tenebrionid beetles [64]). However, salts and clay minerals may remain hydrated. In the Tarapacá Region of the Atacama Desert, gypsum crusts colonized by epilithic lichens and endolithic bacteria benefit from the coastal fog, called ‘camanchaca’, charged with humid air with a relative humidity close to 100% [65]. These frequent fog events [59] result in the continuous deliquescence of halite nodules in local salars, providing constant liquid water to the communities inhabiting the salt rocks [66,67]. This is also the only location in the desert where halite communities harbour the only eukaryote found in this system, a novel *Dolichomastix* alga [68].

The role of fog water inputs in supporting soil microbiomes is much less clear. There is growing evidence that fog capture contributes bioavailable water for cryptic hypolithic communities [69], and supports microbial communities associated with desert plant rhizosheaths [70]. However, with the exception of some specialized plant species such as *S. sabulicola*, where captured fog water is channelled by specially adapted leaf structures down to the root zone [63], fog-derived water inputs are unlikely to penetrate, as liquid, to more than a few millimetres depth in desert pavements.

### 3.4. Groundwater

Even in the driest of deserts, water is present, but not always bioavailable. In cold deserts, permafrost layers, which may exist a few tens of centimetres or a few metres from the desiccated surface, are a potential source of bioavailable water for soil microbiomes [71]. The hydrologically active zone, the horizon above the permafrost that thaws and refreezes with seasonal cycles, provides a saturated soil profile at some distance below the desiccated surface horizon (Figure 1b). In some Antarctic and Arctic regions, such as the Windmill Islands (Ferrari, pers. comm.) and the S*ør* Rondane Mountains in East Antarctica, temperature fluctuations are so high during the summer months that surface soils may be subject to freeze–thaw cycles on a daily basis [72].

**Figure 2:**
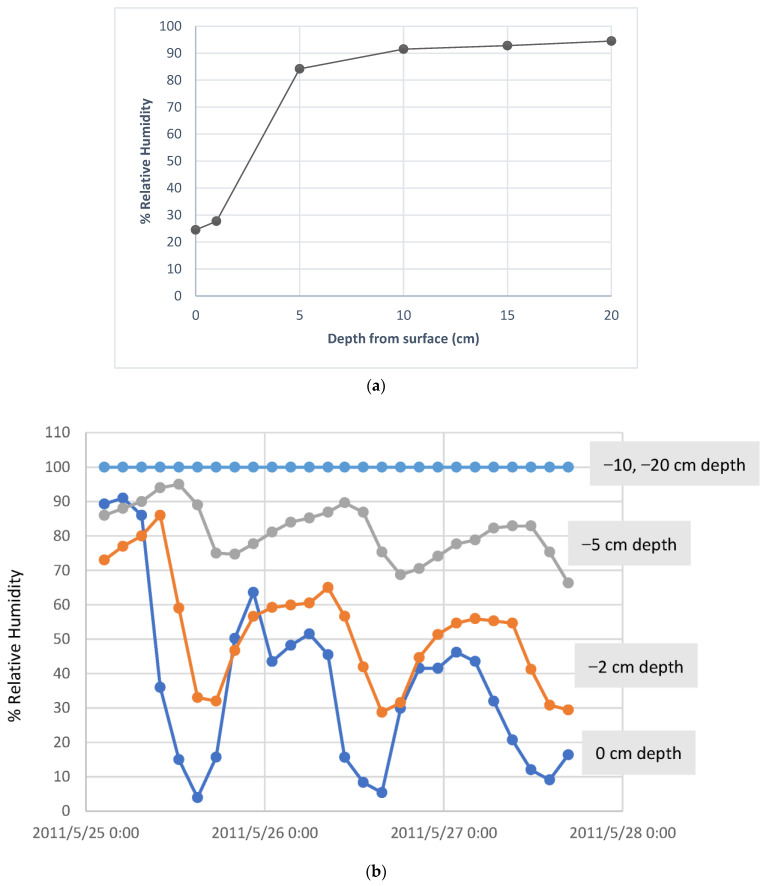

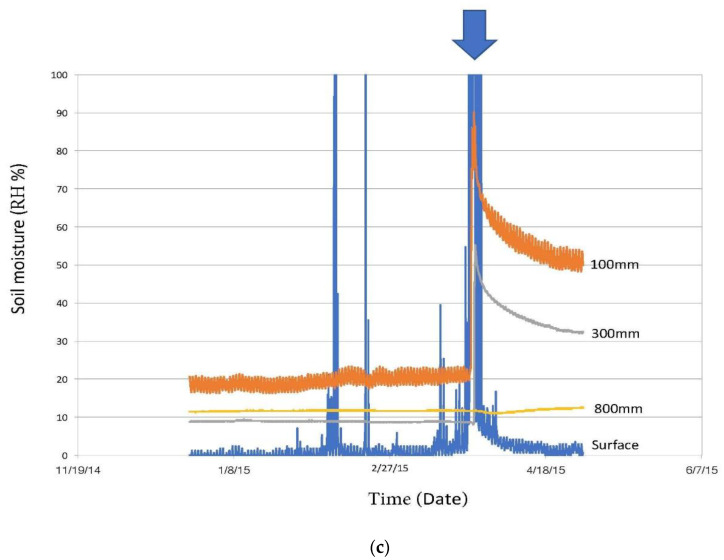
(**a**) Soil %RH values (Thermochron DS1923 iButtons) across a 20 cm depth profile (Miers Valley, East Antarctica, January 2008; (**b**) 86 h iButton (Thermochron DS1923) record of % relative humidity values at specified depths in the soil profile (central Namib Desert, GPS S23.61 E15.17, May 2011); (**c**) temporal variation in soil moisture (%RH) in Atacama Desert soil horizons over a 6-month period, through a major rainfall event (blue arrow). Data from [45].

The presence of a saturated atmosphere at depth and a low surface relative humidity (Figure 2a) will generate a strong thermodynamic driver for the upward diffusion of high relative humidity (RH) water vapour [73,74], potentially available to shallow sub-surface microbial communities (see below). In hot deserts, groundwater may be able to fulfil a similar role, even though such subterranean liquid flows may be found tens or hundreds of metres below ground level. Measurements of soil atmospheric relative humidity values in shallow soil depth profiles in the central (hyper-arid zone) Namib Desert (Figure 2b) and the Atacama Desert (Figure 2c) are strongly suggestive of the upward transport of subsurface water vapour [75], whether derived from deep subterranean groundwater or residual water from infrequent rainfall recharge.

Irrespective of the origins of water vapour in desert soils, nocturnal distillation may also provide a mechanism for generating condensed (liquid) water in the upper soil horizons [40], under conditions where nocturnal soil surface temperatures are sufficiently low to yield measurable increases in shallow sub-surface soil water content [40].

### 3.5. Adsorption of Water from the Atmosphere

It has been well-established that fruticose lichens have the capacity to adsorb water from moist air [76], without the need for condensation processes. The capacity for microorganisms to acquire cellular water directly from the atmosphere (i.e., H_2_O(g)) is not well established, although the permeability of the cell membrane to water molecules and the hypertonicity of the cellular cytoplasm suggest that this process is both physically and thermodynamically feasible. The close correlation between desert soil surface and shallow sub-surface relative humidity values (Figure 2b) suggests that the atmosphere of the near-surface (0 to 2 cm depth) soil horizon is in equilibrium with the above-surface atmosphere, and that water is potentially available to the shallow soil microbial communities that are able to adsorb it. One recent study suggested that, each night, an equivalent of ∼30  μm rainfall may enter the soils of the hyper-arid core of the Atacama Desert via atmospheric water vapour adsorption [77].

Whether microbial cells directly adsorb atmospheric moisture or not, there is good evidence that the biofilm structures in which most soil organisms reside do have this capacity. Most soil microbial communities are embedded in EPS matrices [78], composed of compositionally heterogeneous high-molecular-weight glycan polymers [79] that can constitute up to 90% of the biomass of a biofilm [80]. Such compounds are rich in free hydroxyl (-OH) and amino (-NH_2_) groups, both of which are strongly hydrophilic and contribute to the water-holding capacity of EPSs [81].

In addition, bacterial production and excretion of EPS constituents is strongly stimulated by exposure to stress, particularly sub-lethal heat [82] and osmotic stresses [7], and has been implicated in water retention in desert soil biocrust communities [83]. It has also been demonstrated that the hygroscopic properties of EPSs facilitate both the acquisition and retention of water from the atmosphere [84,85].

Microorganisms accumulate a wide array of low-molecular-weight organic solutes (including monosaccharide and oligosaccharide sugars, polyols, amino acids and their derivatives, ectoines and betaines [86]), at least some of which have been implicated in cellular responses to osmotic (including desiccation) stress [86]. The hygroscopic disaccharide trehalose, which is accumulated intracellularly in many organisms in response to desiccation [87], is capable of adsorbing and retaining water at atmospheric relative humidity values above 50% [88]. This mechanism of water acquisition by desiccated microbial cells is, at least theoretically, feasible in shallow subsurface microbial communities in both hot [89] and cold desert soils (see Figure 2a,b).

### 3.6. Hygroscopic Minerals and Surfaces

Mineral (and other) surfaces can acquire and retain thin films of water [90,91,92] that may exist from >1 mm depth down to molecular monolayers [93]. While the water in thicker surface films, typically acquired because of the presence of hygroscopic salts [94] or solutes [95], is thought to be bioavailable [96], it is uncertain whether thin surface water layers (<3 molecules thick) are in a liquid phase [96].

The presence of minute salt crystals on surfaces, derived from deposited sea spray in coastal deserts, can lead to deliquescence events (i.e., the formation of liquid brine as the salt absorbs water from the atmosphere). The phenomenon of salt deliquescence may be a primary driver for microbial life in much of the Atacama Desert, where cells and communities are typically active in thin layers of brine inside halite rocks [29,97] or in the NaCl-rich subsurface [97].

There is also recent evidence that endolithic microorganisms can access water from hydrated minerals [98]. Cyanobacteria growing as biofilms in gypsum (CaSO_4_.2H_2_O) minerals induce mineral dissolution accompanied by water extraction and the transformation of gypsum to anhydrite (CaSO_4_). Given that many coastal desert soils, such as in the Namib and Atacama Deserts, are gypsum-rich, this phenomenon may be of considerable biological importance.

### 3.7. In Situ Hydro-Genesis

Apart from the various exogenous sources of biologically available water, all microbial cells have the capacity to generate water endogenously. The oxidative heterotrophic metabolism of carbohydrates is hydro-genic (water-generating: C_6_H_12_O_6_ + 6O_2_ = 6CO_2_ + 6H_2_O), although it is unclear what proportion of intracellular water this (and related) metabolic processes may contribute. Oxygen stable-isotope analyses have suggested that, in actively growing cells, 70% of microbial intracellular water may be metabolically derived [99]. Notably, many intracellular metabolic processes are water-generating, including ligation, condensation, polymerization, and related reactions, in addition to oxidative metabolism.

Under desiccated conditions, when many cells may be in an anhydrobiotic state [99,100] with cellular activity limited to basal metabolic processes [100,101,102,103,104], hydro-genesis from carbohydrate metabolism may be limited. However, there is growing evidence that a recently discovered chemotrophic metabolism may even supplement cellular water in desiccated cells. Atmospheric trace gas assimilation, particularly hydrogen oxidation, has been observed in both hot and cold desert soil microbiomes [105,106,107,108,109]. This process, which uses newly described clades of assimilatory Ni–Fe hydrogenases [107] and is present in a wide range of aerobic soil bacterial phyla [108,109], not only provides the energy and reductant needed to support carbon fixation; the process is also hydro-genic (2H_2_ + O_2_ = 2H_2_O). While the quantitative contributions of this process to the cellular water budget have not yet been accurately determined, we have preliminary evidence that the kinetics of this process are remarkably fast, even in desiccated soils and at low temperatures (Belinda Ferrari, unpublished results). H_2_ uptake rates in Antarctic soils (at 10 °C) were estimated at around 140 pmol.h^−1^ g soil_d.w._ [108]. Using this value as an average over a 24 h period, this is equivalent to the addition of 3.4 nmol H_2_O.g soil (0.06 μg H_2_O.day_-1_ g soil). Maximum H_2_ uptake rates, measured for Antarctic Vestfold Hills and Windmill Island soils that have very high abundances of the atmospheric chemotrophic phyla *Actinobacteriota*, *Eremiobacterota* and Ca. Dormibacterota, were recorded as high as 421 nmol.h^−1^ g soil [100], equivalent to approx. 0.2 mg H_2_O.day^−1^ g soil). The scales of these values suggest that the hydro-genic contribution of H_2_ oxidation in soils may not be altogether trivial over diel timescales.

The process of trace-gas-dependent hydro-genesis in microbial cells has the potential to realign current paradigms on the functional status and capacity of desert soil microbiomes. Recent transcriptomic data on microbial cellular function in hyper-arid soils suggest that a subset of microbial taxa retains some functionality under desiccated conditions [110,111]. Similarly, metaproteomic analyses of hyper-arid Antarctic Reeve Hill (Casey Station) soils show evidence of active expression of Ni–Fe hydrogenases and RuBisCO [105].

## 4. Implications of Climate Change

All climate models predict that the world’s major hot desert regions will become hotter and drier [31]. This will directly affect water availability in soils, both from reduced inputs from rainfall, and from a reduction in the P/PET ratio (the aridity index: the ratio of precipitation to evapotranspiration; used in defining the severity of desert ecosystems [31]). The projected consequences of these changes include increased aridification (the expansion of the size of existing dryland and desert areas [112,113] and a transition of existing desert regions to a higher aridity status, e.g., from arid to hyper-arid). Any change in aridity status can have major impacts on both the macrobiology and the microbiology of affected areas, with the loss of the more desiccation- and heat-sensitive species (e.g., [114,115]), a reduction in primary production and soil organic carbon [116], and a reduction in the fixation of atmospheric nitrogen [117]. Both phototrophic and diazotrophic processes, which are crucial in soil nutrient cycling, are known to be very sensitive to cellular water activity [10,101].

Interestingly, under a warming climate regime, polar deserts are likely to become wetter as a result of increased glacial and permafrost melt and increased snowfall [118,119,120] with associated increases in fertility [121,122]. Such changes may lead to challenges for indigenous xerophilic microbiota which may be increasingly out-competed by the colonization and cosmopolitan r-strategists (rapidly growing microbes), resulting in homogenization and loss of biodiversity [123,124]. A recent investigation in the Windmill Islands region in East Antarctica showed that functional bacterial clades were vulnerable to change, with Gradient Forest modelling revealing 10–12% moisture thresholds as environmental tipping points that are likely to result in significant microbial community compositional shifts (Zhang, Wong, Czechowski et al., manuscript under review). Analysis of Antarctic soils sampled over a 14-year period confirmed this prediction, with wetter soils exhibiting higher relative abundances of microbial phototrophs (micro-algae and cyanobacteria) and fewer trace gas chemosynthetic bacteria (*Eremiobacterota*, Ca. Dormibacterota).

Increased water availability in continental Antarctic soils, via enhanced snowmelt and/or glacial melt, is therefore likely to dramatically impact soil microbial diversity and function. Moisture input from glacial meltwater to surface soils in the McMurdo Dry Valleys led to pronounced shifts in bacterial diversity [125] and activity [126]. Similarly, localized melting from persistent snow drifts has been shown to support metabolically active yeasts and filamentous fungi [127].

However, given the adaptability and resilience of microorganisms [128,129], the negative impacts of climate change on soil microbiomes may be less dramatic than on larger organisms such as plant and animal species [130]. In fact, several studies have shown that drought-adapted bacterial and fungal species not only contributed to the overall resilience and stability of microbial communities [131,132], but also enhanced the resilience of the entire ecosystem [133,134,135].

The extent to which climate-induced changes in microbial community ecology will effect changes in function (at the micro-scale) and ecosystem servicing (at the macro-scale) is still a debated topic, in part due to the complexity of the interactions between the key players (fungi, bacteria, archaea, and viruses) within these communities and the ecosystem around them [136,137]. Such changes are, however, unlikely to be trivial [123]. Nonetheless, we believe that the concept of ‘where there is water, there is life’ remains an important paradigm. It is evident, however, that climate-induced changes in water availability are regionally highly heterogeneous, and the impacts on hot and cold deserts are likely to be very different, if not, in some instances, directly opposite.

## Data Availability

Not applicable.
